# Trends in Hallucinogen-Related Emergency Department and Hospital Admissions, 2016 to 2023

**DOI:** 10.1001/jamanetworkopen.2025.43453

**Published:** 2025-11-13

**Authors:** Jacob T. Steinle, Lisa Gong, Joanna L. Buss, Suraj Shankar, Joshua S. Siegel, Leopoldo J. Cabassa, Ruth Ling, Regina Huang, Danielle R. Adams, Patricia Cavazos-Rehg, Arpana Agrawal, Ginger E. Nicol, Richard Grucza, R. J. Waken, Kevin Y. Xu

**Affiliations:** 1 Department of Psychiatry, Washington University School of Medicine, St Louis, Missouri; 2 Department of Psychiatry, NYU Grossman School of Medicine, New York, New York; 3 Nathan S. Kline Institute for Psychiatric Research, Orangeburg, New York; 4 Center for Mental Health Services Research, George Warren Brown School of Social Work, Washington University, St Louis, Missouri; 5 Center for Holistic Interdisciplinary Research in Psychedelics, Washington University, St Louis, Missouri; 6 Advanced Health Data Institute, Department of Health and Clinical Outcomes Research and Department of Family and Community Medicine, St Louis University, St Louis, Missouri; 7 School of Social Work, College of Health Sciences, University of Missouri, Columbia; 8 Institute for Informatics, Data Science & Biostatistics, Washington University School of Medicine, St Louis, Missouri; 9 Washington University School of Public Health, St Louis, Missouri; 10 Center for Advancing Health Services, Policy and Economics Research, Division of Cardiology, Department of Medicine, Washington University School of Medicine, St Louis, Missouri

## Abstract

This cohort study evaluates trends in emergency department (ED) visits and hospitalizations related to hallucinogen use in the United States.

## Introduction

Hallucinogen use in the United States has increased in recent years, particularly among young adults, amid shifting cultural attitudes and medicalization.^1,2,3,4,5^ US Food and Drug Administration (FDA) breakthrough therapy designations for psilocybin and methylenedioxy-methylamphetamine (MDMA), along with widespread media coverage of their therapeutic potential, coincide with evolving policy landscapes (eg, municipal and state-level decriminalization and legalization).^4^ Together, these developments may be shaping national trends in ways that extend beyond localized policies. Additionally, growing cross-jurisdictional travel to areas with more permissive psychedelic policies^6^ raises questions about whether local reforms may have national implications. It remains unclear whether these evolving cultural, medical, and policy contexts in the United States are reflected in detectable changes in hallucinogen-related emergency department and inpatient admissions at the population level.

## Methods

We conducted this retrospective cohort study using the Merative MarketScan Commercial and Multi-State Medicaid databases (2016-2023), including individuals aged 16 to 64 years. Monthly rates of hallucinogen-related emergency or hospital admissions (*ICD-10* code F16xx) were calculated as a proportion of all substance-related admissions (*ICD-10* codes F10xx to F19xx, excluding nicotine). Temporal trends were estimated using bayesian multiple change-point (MCP) models (eMethods in Supplement 1). Analyses used SAS version 9.4 (SAS Institute) and R version 4.5.1 (R Project for Statistcal Computing), with the mcp package. The study followed RECORD-PE guidelines and was exempt from human participant review by the Washington University human research protection office.

## Results

**Figure 1.**
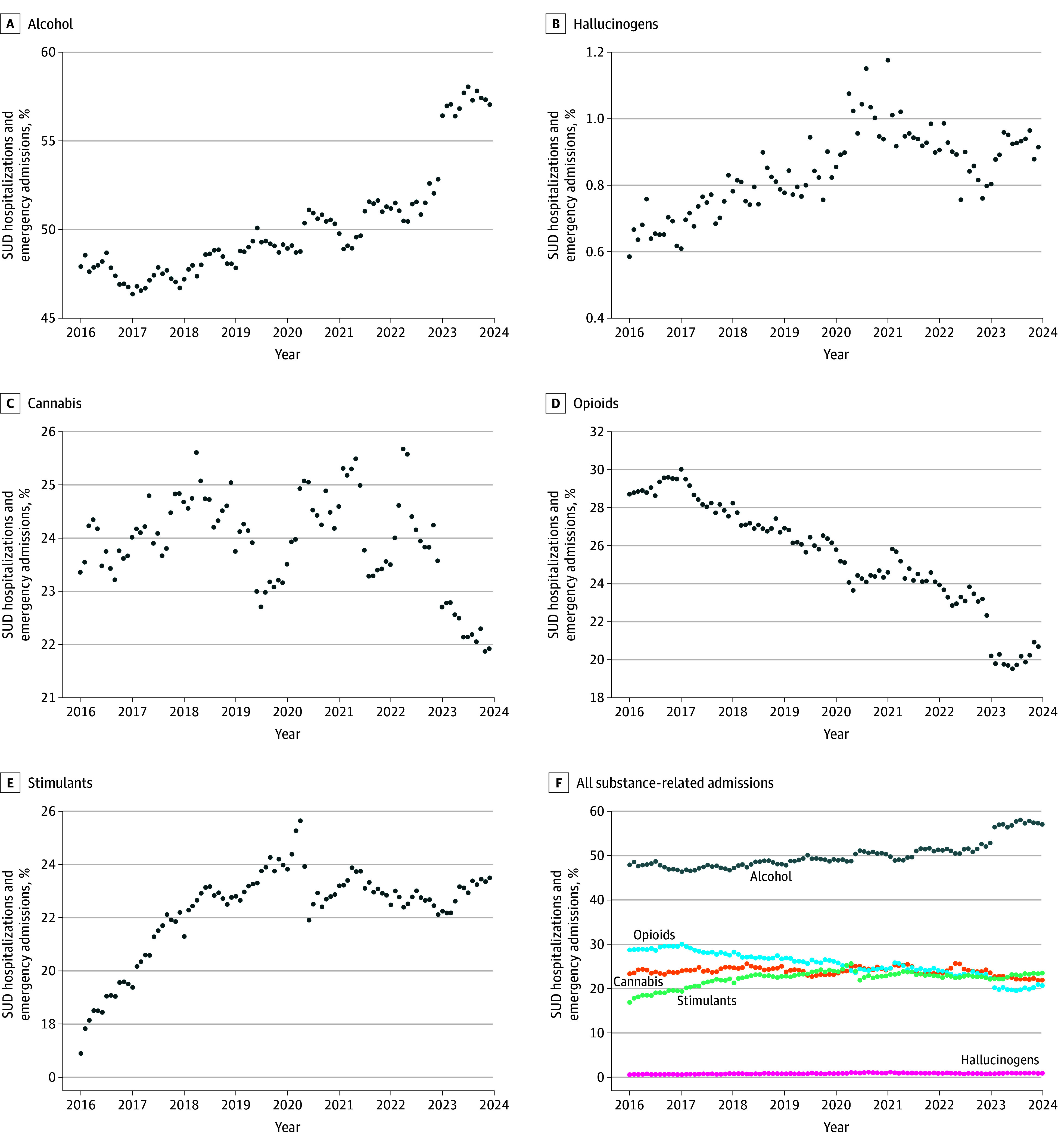
Comparison of Monthly Emergency Department or Inpatient Substance-Related Admission Trends for Different Drug Types in the United States, 2016 to 2023 Each panel displays the monthly percentage of all substance-related emergency department visits and hospitalizations (*ICD-10* codes F10xx to F19xx, excluding nicotine) involving a specific substance category. Substance-related diagnoses were identified in any diagnostic position, and categories are not mutually exclusive, as individual admissions may involve multiple substances. The substances shown include alcohol (range, 46.4%-58.0%), hallucinogens (range, 0.6%-1.2%), cannabis (range, 21.9%-25.7%), opioids (range, 19.5%-30.0%), and stimulants (range, 16.9%-25.6%). Each substance is shown in a separate panel with an independent y-axis scale to allow visualization of temporal patterns despite differences in absolute magnitude across substance categories. SUD indicates substance use disorder.

**Figure 2.**
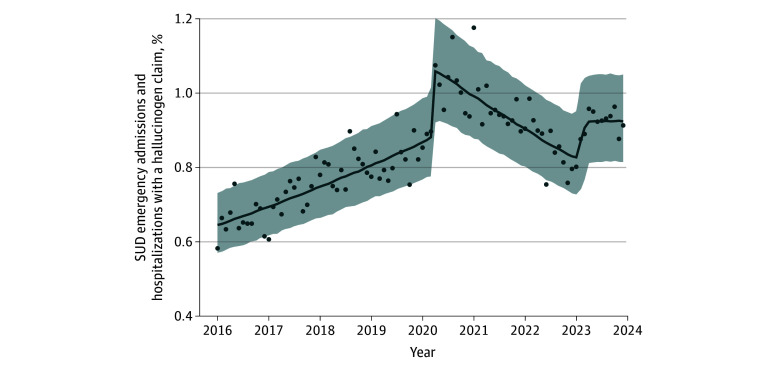
Bayesian Multiple Change Point Analyses Evaluating the Monthly Proportion of Hallucinogen-Related Admissions Among Substance-Related Emergency Department and Inpatient Admissions in the United States, 2016 to 2023 This figure presents the monthly percentage of substance-related admissions involving hallucinogens, with results from bayesian multiple change-point analysis. Two change-points were identified: April 2020 and March 2023. From January 2016 to April 2020, monthly rates increased by 0.62% (95% CI, 0.49%-0.74%) per month. From April 2020 to March 2023, rates declined by 0.76% (95% CI, −0.99% to −0.48%) per month. No significant trend was observed from March through December 2023. SUD indicates substance use disorder.

Among 1 355 161 individuals with substance-related admissions from 2016 to 2023, 21 700 (1.6%; median [IQR] age, 28 [21-39] years) had at least 1 hallucinogen-related admission. Of these individuals, 13 079 (60.3%) had Medicaid coverage, and among those with race and ethnicity data (Medicaid enrollees), 487 (4.2%) were Hispanic; 5412 (47.1%) non-Hispanic Black; and 5064 (44.1%) non-Hispanic White. Across individuals with hallucinogen-related admissions with at least 6 months of continuous enrollment preadmission (15 420 individuals), 6-month preadmission comorbidities included mood disorders (5394 [35.0%]), anxiety disorders (4500 [29.2%]), schizophrenia-spectrum disorders (2262 [14.7%]), and non-nicotine substance–related disorders (2913 [18.9%], including opioids [n = 894], alcohol [n = 1048], cannabis [n = 1487], stimulants [n = 905], and sedatives [n = 182]). As shown in Figure 1, the monthly share of hallucinogen-related admissions ranged from 0.59% in January 2016 to 1.18% in January 2021 of substance-related admissions throughout the 7-year period. By contrast, alcohol- and opioid-related encounters accounted for the overwhelming majority of admissions throughout the study period (Figure 1). As illustrated in Figure 2, MCP models identified rising hallucinogen-related admissions until early 2020, followed by a decline through 2023.

## Discussion

It is well documented that self-reported hallucinogen use is rising in the United States^1,3,5^; however, emergency and hospital admissions involving hallucinogens remain relatively rare. Our analysis identified rising admissions until early 2020, followed by a decline through 2023, with patterns stabilizing slightly above prepandemic levels. These segmented trends parallel California data showing a 2020 peak followed by a decline,^2^ raising questions about a possible pandemic-related disruption of an upward trajectory.

Hallucinogens continue to account for a small fraction of substance-related hospital admissions, outweighed by alcohol- and opioid-related encounters. By expressing hallucinogen-related admissions as a proportion of all substance-related admissions, we provide a standardized measure of their relative contribution to acute morbidity. Absolute rates offer a complementary perspective but are difficult to estimate reliably with claims data,^2,5^ given variation in enrollment and health care utilization.

Study limitations include unfamiliarity with *ICD-10* hallucinogen coding among clinicians compared with alcohol or opioids, likely leading to underascertainment. F16 codes aggregate diverse substances, including classical hallucinogens, ketamine, and esketamine (FDA approved in 2019), precluding separation of their individual contributions. Additionally, our dataset lacks geographic identifiers, limiting assessment of localized policy effects, and temporal trends likely reflect multiple overlapping influences, making it difficult to attribute inflection points to specific events. These findings invite consideration of whether observed patterns of hallucinogen-related admissions correspond with current Schedule I classification, recognizing that low prevalence does not imply absence of risk. As use patterns and regulatory environments continue evolving, ongoing surveillance remains critical.

## Notes

Supplement 1. **eMethods. **Protocol for Statistical Analysis

Supplement 2.
Data Sharing Statement

## References

[1] Livne O, Shmulewitz D, Walsh C, Hasin DS. Adolescent and adult time trends in US hallucinogen use, 2002-19: any use, and use of ecstasy, LSD and PCP. Addiction. 2022;117(12):3099-3109. doi:10.1111/add.15987 35978453 PMC9994631

[2] Garel N, Tate S, Nash K, Lembke A. Trends in hallucinogen-associated emergency department visits and hospitalizations in California, USA, from 2016 to 2022. Addiction. 2024;119(5):960-964. doi:10.1111/add.16432 38213013

[3] Walsh CA, Livne O, Shmulewitz D, Stohl M, Hasin DS. Use of plant-based hallucinogens and dissociative agents: US time trends, 2002-2019. Addict Behav Rep. 2022;16:100454. doi:10.1016/j.abrep.2022.100454 36119808 PMC9471967

[4] Siegel JS, Daily JE, Perry DA, Nicol GE. Psychedelic drug legislative reform and legalization in the US. JAMA Psychiatry. 2023;80(1):77-83. doi:10.1001/jamapsychiatry.2022.4101 36477830 PMC10069558

[5] Rockhill KM, Black JC, Ladka MS, . The rise of psilocybin use in the United States: a multisource observational study. Ann Intern Med. 2025;178(7):1052-1054. doi:10.7326/ANNALS-24-03145 40258279

[6] Black JC, Rockhill KM, Fox EJ, Jewell JS, Dart RC, Monte AA. Psychedelic trips: travel within the United States to Use psychedelic drugs after legalization. Ann Emerg Med. Published online June 30, 2025. doi:10.1016/j.annemergmed.2025.05.016 40590826

