# Supplementary material for: Trends in Hallucinogen-Related Emergency Department and Hospital Admissions, 2016 to 2023
Source: JAMA Netw Open. 2025 Nov 13;8(11):e2543453. doi: 10.1001/jamanetworkopen.2025.43453 (PMC12616458; doi:10.1001/jamanetworkopen.2025.43453)
Supplement: Supplement 1. — eMethods. Protocol for Statistical Analysis [file jamanetwopen-e2543453-s001.pdf]

Supplemental Online Content

Steinle JT, Gong L, Buss JL, et al. Trends in Hallucinogen-Related Admissions, 2016 to 2023. *JAMA Netw Open*. 2025;8(11):e2543453. doi:10.1001/jamanetworkopen.2025.43453

eMethods. Protocol for Statistical Analysis

This supplemental material has been provided by the authors to give readers additional information about their work.

© 2025 Steinle JT et al. *JAMA Network Open*.

eMethods. Protocol for Statistical Analysis

Justification for MCP models: Given rising hallucinogen use since the mid-2010s,[1] we used Bayesian multiple change-point (MCP) models to assess trends in hallucinogen-related emergency admissions and hospitalizations from January 2016 to December 2023. Because psychedelic decriminalization began in 2020 but varied by state/municipality and overlapped with the COVID-19 pandemic, MCP models were chosen over methods like interrupted time series, which require a single predefined time point for comparison.

Derivation of the cohort: We identified 1,355,161 individuals in the US, aged 16-64 years, with ≥1 substance-related emergency admission or hospitalization during enrollment, defined by ≥1 claim for ICD-10 codes F10.XX–F19.XX (excluding nicotine-related disorders) in acute care settings (emergency departments, inpatient hospitals, or ambulances, per the MarketScan STDPLAC variable), with events separated by ≥7 days. Within this group, 21,700 had ≥1 hallucinogen-related admission (F16.XX). Continuous enrollment was not required, as analyses used monthly aggregate rates. Data linkage, de-identification, cleaning, adjudication, and quality control audits are overseen by Merative (formerly IBM Watson).

Variables: The independent variable was time, measured in 84 monthly intervals from January 2016 to December 2023. We used monthly rather than annual rates to capture rapid fluctuations in drug-related outcomes.[2–4] Because the MarketScan population size varies over time, we normalized hallucinogen-related admissions as a percentage of all monthly substance-related emergency admissions or hospitalizations (*ICD-10* F10.XX–F19.XX, excluding nicotine-related disorders). The dependent variable was the monthly percentage of hallucinogen-related admissions (F16.XX) with its standard errors. We assessed comorbidities in the 6 months preceding the index (first) hallucinogen-related admission using the following *ICD-10* codes: mood-related disorders (F30.XX-F39.XX), anxiety-related disorders (F40.XX-F49.XX), schizophrenia spectrum disorders (F20.XX-F29.XX), and substance-related disorders (F10.XX-F19.XX; nicotine codes excluded). Specific substance categories included hallucinogen-related disorders (F16.XX), sedative-related disorders (F13.XX), stimulant-related disorders including cocaine and methamphetamine (F12.XX, F15.XX), cannabis-related disorders (F12.XX), alcohol-related disorders (F10.XX), and opioid-related disorders (F11.XX).

Analytic Procedure: We first described demographic characteristics and psychiatric comorbidities during the 6 months before the first hallucinogen- related admission among 12,830 individuals with ≥6 months of continuous enrollment. To model rare events, we fitted linear models to log-transformed rates, which perform better than logistic models under sparse conditions. Change points were identified using Bayesian MCP models, which assume that outcomes follow a stable distribution within intervals but shift in mean, variance, or autocorrelation at change points. To account for seasonal variation in substance-related admissions,[5-7] we included sine and cosine terms in the MCP models. [8] Change points were initialized based on visual inspection but estimated using default uniform priors across all time points. Models used 4,000 Markov chain Monte Carlo (MCMC) iterations in three chains, with the first 1,000 discarded as burn-in, yielding 9,000 posterior draws. We back-transformed MCMC estimates to the original data scale for interpretation.

We sequentially fitted models with increasing numbers of change points, and used a combination of model fit diagnostics and model comparison tools to define our stopping rule. Specifically, we use Leave-One-Out Cross Validation (LOO-CV) via Pareto-smoothed importance sampling[9] to compare the predictive performance of models, and a combination of

© 2025 Steinle JT et al. *JAMA Network Open*.

large Gelman-Rubin convergence diagnostic (R-hat>1.05) and Pareto k estimates (k-hat>0.7)[9-10] as diagnostic tools that may indicate poor model fit. If either a) model diagnostics indicate poor fit, or b) the standardized difference in the estimated log predictive density (ELPD) between two models is not large ($abs(\hat{E}LPD/seELPD)$ < 1.96), we stopped and used the previous, simpler model. Based on these criteria, the log-linear regression model with two change points was selected as the best fit to these data. This model was parsimonious with 12 parameters and had no evidence of poor model fit or convergence issues (Pareto k-hat < 0.7, R-hat < 1.05 for all parameters). The mean percentage change was estimated with 95% confidence intervals (CIs) by segment between change points.

Recognizing that hallucinogen-related diagnoses may reflect unrelated primary reasons for care (e.g., COVID-19, depression), we conducted robustness checks that limited the sample to people with inpatient admissions where a hallucinogen-related disorder was in the primary diagnostic position (n=1,311 individuals). This distinction was possible for inpatient data but not for emergency department admissions, which lack primary diagnosis coding in MarketScan. The sensitivity analysis yielded similar temporal patterns to our main findings, with hallucinogen-related admissions increasing by 0.62% per month from January 2016 to April 2020 (95% CI: 0.49% to 0.74%), declining by 0.76% per month from April 2020 to March 2023 (95% CI: -0.99% to -0.48%), and remaining stable thereafter with a 0.01% monthly change from March 2023 to December 2023 (95% CI: -0.38% to 0.53%). Monthly rates for primary hallucinogen-related admissions ranged from 0.14% to 0.50% across the observation period. All analyses were conducted using SAS 9.4 and R package mcp.[11]

REFERENCES

1. Livne O, Shmulewitz D, Walsh C, Hasin DS. Adolescent and adult time trends in US hallucinogen use, 2002-19: any use, and use of ecstasy, LSD and PCP. *Addiction.* 2022;117(12):3099-3109.
2. Parks RM, Rowland ST, Do V, et al. The association between temperature and alcohol- and substance-related disorder hospital visits in Ne York State. *Commun Med (Lond).* 2023;3(1):118.
3. Mejldal A, Sogaard Nielsen A, Wallhed Finn S. Seasonal Variations in Treatment Seeking for Alcohol Use Disorder in the Total Danish Population. *J Stud Alcohol Drugs*. 2024;85(5):674-681.
4. Batts K, Pemberton M, Bose J, et al. Comparing and Evaluating Substance Use Treatment Utilization Estimates from the NSDUH and Other Data Sources. 2014 Apr. In: CBHSQ Data Review. Rockville (MD): Substance Abuse and Mental Health Services Administration (US).
5. Zhang HX, Khan A, Chen Q, Larsson H, Rzhetsky A. Do psychiatric diseases follow annual cyclic seasonality? *Plos Biol.* 2021;19(7).
6. Lewer D, Brothers TD, Gasparrini A, Strang J. Seasonal, weekly and other cyclical patterns in deaths due to drug poisoning in England and Wales. *Addiction.* 2023;118(8):1596-1601.
7. Goedel WC, Marshall BDL, Spangler KR, et al. Increased Risk of Opioid Overdose Death Following Cold Weather: A Case-Crossover Study. *Epidemiology.* 2019;30(5):637-641.
8. Cleveland RB, Cleveland WS, McRae JE, Terpenning I. 1990. Stl: A seasonal-trend decomposition procedure based on loess. *J Official Statistics*, 6(1):3–73.
9. Vehtari A, Gelman A, Gabry J. Practical Bayesian model evaluation using leave-one-out cross-validation and WAIC (vol 27, pg 1413, 2017). *Stat Comput.* 2017;27(5):1433-1433.
10. Gelman A, Rubin DB. Inference from iterative simulation using multiple sequences. *Stat science.* 1992 Nov;7(4):457-72.
11. Lindeløv JK. mcp: An R package for regression with multiple change points. Accessed

April 19, 2025. https://osf.io/preprints/fzqxv/.
