# Supplementary material for: Trends in Hallucinogen-Related Emergency Department and Hospital Admissions, 2016 to 2023
Source: JAMA Netw Open. 2025 Nov 13;8(11):e2543453. doi: 10.1001/jamanetworkopen.2025.43453 (PMC12616458; doi:10.1001/jamanetworkopen.2025.43453)
Supplement: Supplement 2. — Data Sharing Statement [file jamanetwopen-e2543453-s002.pdf]

Data Sharing Statement

Steinle. Trends in Hallucinogen-Related Emergency Department and Hospital Admissions, 2016 to 2023. *JAMA Netw Open*. Published November 13, 2025.
doi:10.1001/jamanetworkopen.2025.43453

Data
Data available: No

Additional Information
Explanation for why data not available: The data is proprietary and can be accessed via a request to Merative (www.merative.com).
